# Treatment of advanced gastrointestinal cancer with genetically modified autologous mesenchymal stem cells - TREAT-ME-1 - a phase I, first in human, first in class trial

**DOI:** 10.18632/oncotarget.20964

**Published:** 2017-09-16

**Authors:** Jobst C. von Einem, Sylvia Peter, Christine Günther, Hans-Dieter Volk, Gerald Grütz, Christoph Salat, Oliver Stoetzer, Peter J. Nelson, Marlies Michl, Dominik P. Modest, Julian W. Holch, Martin Angele, Christiane Bruns, Hanno Niess, Volker Heinemann

**Affiliations:** ^1^ Department of Medical Oncology and Comprehensive Cancer Center, University Hospital Grosshadern, LMU, Munich, Germany; ^2^ Apceth GmbH & Co. KG, Munich, Germany; ^3^ Institute for Medical Immunology and Berlin-Brandenburg Center for Regenerative Medicine, Charité-Universitätsmedizin, Berlin, Germany; ^4^ Haemato-Onkologische Schwerpunktpraxis Prof. Salat, Dr. Stoetzer, Munich, Germany; ^5^ Department of Medicine IV, University Hospital of Munich, LMU, Munich, Germany; ^6^ Department of Surgery, University Hospital Grosshadern, LMU, Munich, Germany; ^7^ General, Visceral and Cancer Surgery, University Hospital of Cologne, Cologne, Germany

**Keywords:** mesenchymal stem cells, gastrointestinal cancer, CCC, CRC, GDEPT

## Abstract

**Purpose:**

This phase I, first in human, first in class clinical study aimed at evaluating the safety, tolerability and efficacy of treatment with genetically modified mesenchymal stromal cells (MSC) in combination with ganciclovir (GCV). MSC_apceth_101 are genetically modified autologous MSCs used as vehicles for a cell-based gene therapy in patients with advanced gastrointestinal adenocarcinoma.

**Experimental design:**

The study design consisted of a dose-escalation 3 + 3 design. All patients (*n* = 6) were treated with up to three applications of MSC_apceth_101, followed by GCV infusions given on three consecutive days starting 48 hours after injection of MSC_apceth_101. Three of six patients received a total dose of 1.5 × 10^6^ cells/kg. Two patients received three doses of 1 × 10^6^ cells/kg, while one patient received only two doses of 1 × 10^6^ cells/kg due to a SADR.

**Results:**

Six patients received MSC_apceth_101. No IMP-related serious adverse events occurred. Adverse-events related to IMP-injection were increased creatinine, cough, fever, and night sweat. TNF, IL-6, IL-8, IL-10 and sE-Selectin, showed that repeated application is immunologically safe, but induces a switch of the functional properties of monocytes to an inflammatory phenotype. Treatment induced stable disease in 4/6 patients, and progressive disease in 2/6 patients.

**Conclusion:**

Treatment with MSC_apceth_101 in combination with GCV demonstrated acceptable safety and tolerability in patients with advanced gastrointestinal adenocarcinoma.

## INTRODUCTION

In patients with end-stage gastrointestinal (GI) cancer, who have acquired resistance to the available cytotoxic agents and biologicals, overall survival (OS) is typically in the range of few months [1–4]. Therefore, there is a clear medical need for innovative treatments options.

Adenocarcinoma of the GI- and Hepato-Pancreato-Biliary (HPB-) tract usually share the morphological characteristic of a tumor-supporting stroma. The cells that make up the stroma are assumed to mainly consist of non-malignant cells that have become encroached by the tumor. They include immune cells, fibroblasts, endothelial cells and pericytes [5]. In this complex microenvironment, stromal and cancer cells interact reciprocally with both tumor enhancing and inhibitory effects as result of these interactions. The main pro-tumorigenic effect of the stroma probably lies in organizing “tumor infrastructure”, which consists of blood vessels and connective tissue. Thereby stromal cells aid in supplying tumor cells with oxygen and nutrients and thus are mandatory for proliferating tumor cells to develop beyond a microscopic state and overcome tumor dormancy [6]. Conversely, evidence from pancreatic cancer mouse models points towards tumor-restraining aspects of the stroma [7, 8]. Regardless of this controversy however, the fact that most tumors share the commonality of constructing a stromal compartment in the process of growing, encourages one to develop strategies that exploit this aspect by channeling therapeutics into the tumor *via* this route. Hence, this therapy should ideally be universally effective in adenocarcinoma of different origin, irrespective of the genetic phenotype and possibly acquired resistance mechanisms to conventional therapy.

Mesenchymal stem/stromal cells (MSCs) are precursor cells, which can be isolated and expanded easily in large amounts from adult mammals [9]. Important physiological functions of MSCs are their participation in niche-formation (e.g. for hematopoietic stem cells in the bone marrow) and tissue regeneration [10]. Within their physiological functions, MSCs display broad capabilities of (trans-) differentiation, immune-evasion, immunomodulation, and trophic factor secretion [11–13]. Furthermore, as numerous preclinical studies suggest, MSCs represent a major precursor population for cells of the tumor stroma (i.e. tumor-associated fibroblasts (TAF), pericytes, and endothelial cells), thus making MSCs an ideal vehicle cell for delivery of tumor-directed therapy (reviewed in [14]).

Several preclinical animal studies conducted by us and others have successfully confirmed tumor homing of genetically modified MSCs from the circulation and shown efficacy of MSC-based therapies [15–20]. The transgenes inserted into MSCs prior to injection usually enable these cells either to secrete proteins with direct or indirect inhibitory function on tumor cells, or encode for an enzyme that specifically allows these cells to turn an otherwise non-toxic prodrug into its toxic form. The latter strategy, which has been utilized by our group, is termed “Gene-directed enzyme-producing therapy (GDEPT)”, or simply “suicide-gene therapy” (reviewed in [21]). Herpes-simplex-virus thymidine kinase (HSV-TK), which catalyzes the phosphorylation of the prodrug GCV to the toxic compound ganciclovir triphosphate, is one of the most commonly deployed suicide genes. Phosphorylated GCV inhibits DNA polymerases and thereby induces apoptosis. Advantages of GDEPT strategies lie in the high bioavailability, permeability, and half-life of the prodrug as compared to most conventional chemotherapies. The toxic metabolites diffuse to, are actively transported through gap junctions, and are taken up *via* phagocytosis by surrounding cells. This “bystander effect” leads to creation of a toxic environment that ultimately not only kills the suicide gene carrying cells but also many of the surrounding tumor and stromal cells [22–24].

Here, we report the application of autologous human MSCs genetically modified to express HSV-TK (investigational medicinal product (IMP): MSC_apceth_101) for the treatment of GI-adenocarcinoma in a phase I clinical trial (TREAT-ME-1 trial). To our knowledge, this is the first clinical study ever to investigate the use of genetically modified MSCs in humans. The primary objective of this phase I study was to assess safety and tolerability of the product MSC_apceth_101. Secondary objectives were (a) tumor response, measured by total and individual size of local relapse or metastases by CT or MRI according to RECIST criteria and by tumor and serum markers, (b) time to progression up to 1 year after first MSC_apceth_101 administration and (c) overall survival up to 1 year after first MSC_apceth_101 administration. Furthermore, the study addressed the feasibility of the novel treatment approach.

## RESULTS

### Patient enrollment and characteristics

Between November 2013 and December 2014, six patients were enrolled in the study. Three male patients suffered from metastatic colorectal cancer (mCRC), two female patients from Pancreatic cancer (PanCa) and one female patient from a cholangiocarcinoma (CCC). All patients were heavily pretreated. Detailed patient characteristics and side effects are provided in Tables 1 and 2. Three of the six patients received the cells at a total dose of 1.5 × 10^6^ cells/kg. The following two patients received three doses of 1 × 10^6^ cells/kg adding up to a total dose of 3 × 10^6^ cells/kg, while one patient only received two doses due to a severe adverse drug reaction (SADR).

**Table 1:** Baseline patient characteristics

Patient Characteristics									
Patient Number	Sex	Age	Race	Neoplasm	Time since diagnosis (years)	Metastases	TNM	ECOG	No. of prior Chemo
1	female	49	Caucasian	CCC	6	Liver	T3N1Mx	1	7
2	male	79	Caucasian	mCRC	5	Liver, Lung, Lymph node	T3N1M1	2	7
3	female	71	Caucasian	PancCa	3	Lung,	T3N1Mx	1	5
4	male	63	Caucasian	mCRC	3	Liver, Lymph node	T2NxM1	0	2
5	male	76	Caucasian	mCRC	1	Abdomen, Lymphnode	T4N2M1	1	4
6	female	54	Caucasian	PanCa	1	Liver, Lung, Lymph node	T3N1M1	1	3

**Table 2:** Incidence of Adverse Events (AEs), Serious Adverse Events (SAEs) and Serious Adverse Drug Reactions (SADR)

INCIDENCE OF AEs	LOW DOSE (n=3) 1.5 × 10^6^ cells/kg	HIGH DOSE (n=3) 3 × 10^6^ cells/kg
**Total No. AEs**	**18**	**28**
**Total No. SAEs (marked with** ^*^)	**5**	**2**
**Total No. SADR (marked with** ^§^)	**0**	**1**
**Blood and lymphatic system disorders**		
∙ Anemia	0	2
**Gastrointestinal disorders**		
∙ Abdominal pain	3	1
∙ Gastrointestinal hemorrhage	0	1*^§^
∙ Constipation	1*	0
∙ Diarrhea	1*	2
∙ Mucous stools	1	0
∙ Nausea	1	1
∙ Vomiting	1	1
**General disorders and administration site conditions**		
∙ Asthenia	0	2 (1*)
∙ Device dislocation*	1*	0
∙ Fatigue	1	1
∙ General physical health deterioration	0	1
∙ Pyrexia	1	2
**Hepatobiliary disorders**		
∙ Cholangitis	1*	0
∙ Cholestasis	1*	0
**Investigations**		
∙ Blood Creatinine increased	0	1
∙ Blood fibrinogen increased	0	1
∙ Creatinine renal clearance decreased	0	1
∙ ECOG performance status worsened	2	1
∙ Hepatic enzyme increased	0	1
∙ Inflammatory marker increased	1	0
∙ International normalized ratio increased	0	1
**Metabolism and nutrition disorders**		
∙ Catabolic state	0	1
∙ Malnutrition	0	1
**Musculoskeletal and connective tissue disorders**		
∙ Growing pains	0	1
**Nervous system disorders**		
∙ Dizziness	0	1
**Renal and urinary disorders**		
∙ Bladder Pain	0	1
**Respiratory, thoracic and mediastinal disorders**		
∙ Cough	0	1
∙ Dyspnea	1	0
**Skin and subcutaneous tissue disorders**		
∙ Dry skin	1	0
∙ Night sweats	0	1
**Vascular disorders**		
∙ Hypertension	0	1

### Safety and tolerability

Overall, the incidence of IMP-related adverse events was low. Table 2 presents all Serious Adverse Drug Reactions (SADR), Serious Adverse Events (SAE) and adverse events (AE).

No SAE with a causal relationship to the IMP was reported in the six patients. The following non-serious AE causally related to MSC_apceth_101 were documented: increased levels of creatinine (maximum 1.5 mg/dl-upper normal level: < 1.2 mg/dl), cough, fever and night sweat. No acute and long-term safety concerns occurred.

As far as GCV is concerned, gastrointestinal hemorrhage was reported in one out of six patients as an SAE (prolonged hospitalization) with a causal relationship. This SADR led to the avoidance of the third dose in one patient. This patient suffered from a mild diarrhea on the first day of GCV administration. These symptoms were self-limiting within two days. About 24 hours after the second MSC_apceth_101 infusion, a second episode of mild diarrhea started. On the same day, antibiotic therapy with amoxicillin/clavulanic acid was applied because of fever. One day later, the patient received GCV as usual over 3 days and in parallel continued amoxicillin/clavulanic acid. On the last day of the GCV infusion, perianal bleeding was observed for the first time. Amoxicillin/clavulanic acid and GCV were therefore stopped. Despite the use of CT and endoscopy no clear cause for this event could be detected. Whether GCV contributed to this hemorrhage could not be excluded. Nevertheless, since gastrointestinal hemorrhage was not reported in the summary of product Characteristics (SmPC) of intravenously applied ganciclovir (Cymeven^®^), the event was reported as a SADR.

The following non-serious adverse events had also a causal relationship to GCV: abdominal pain, fever, and raised creatinine. Significant changes in arterial oxygen saturation were not detectable. Quality of life (QoL), evaluated *via* state-of-the art questionnaires (QLQ-C30), was good and showed that study-related reduction in QoL was not reported.

### Immunological biomarkers

We addressed the following five questions: i) Does the MSC treatment triggers an inflammatory response which can be systemically detected? A temporary 2-3-fold mild increase of circulating IL-6 plasma levels was observed in two of three patients receiving high-dose MSCs, but not in the three patients treated at low-doses, and were seen immediately after first MSCs application reaching basic levels within 3 days. Repeated dosing did not induce further IL-6 elevations. TNF, IL-8, and IL-10, as well as sE-Selectin levels did not increase in response to therapy. ii) Does MSC therapy induce endothelial cell (EC) activation? We did not observe any increase of total IL-8 or soluble E-selectin as marker of EC activation. iii) Does MSC therapy activates T cells to improve anti-tumor response? The most sensitive parameter for monitoring global T-cell activation, the rise of monocytic HLA-DR expression, did not show significant changes during observation period. iv) Does MSC therapy reprogram monocytes/macrophages to a stronger M1 answer with improved anti-tumor responsiveness? The pro-inflammatory potency (*ex vivo* TNF release) of peripheral blood monocytes following Toll-like Receptor (TLR)-4 stimulation increased over the observation time while the anti-inflammatory potency (IL-10 release) decreased. This resulted in an enhanced TNF/IL-10 ratio in four patients analyzed (mean: 3.73 fold, range 1,32 - 6,01 fold) (Figure 2). v) Are gene-modified MSC induce immunogenicity? No T-cell sensitization in response to the repeated application of autologous gene-modified MSCs as measured by the sensitive IFN-g Elispot was observed. In addition, there were no changes in monocytic HLA-DR expression, known as a sensitive immune competence marker.

**Figure 1:**
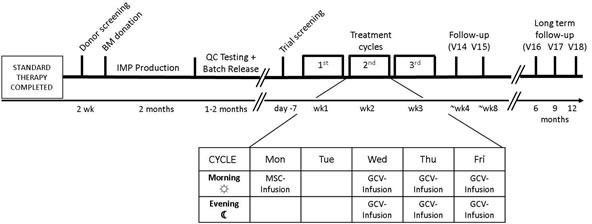
Application scheme

**Figure 2:**
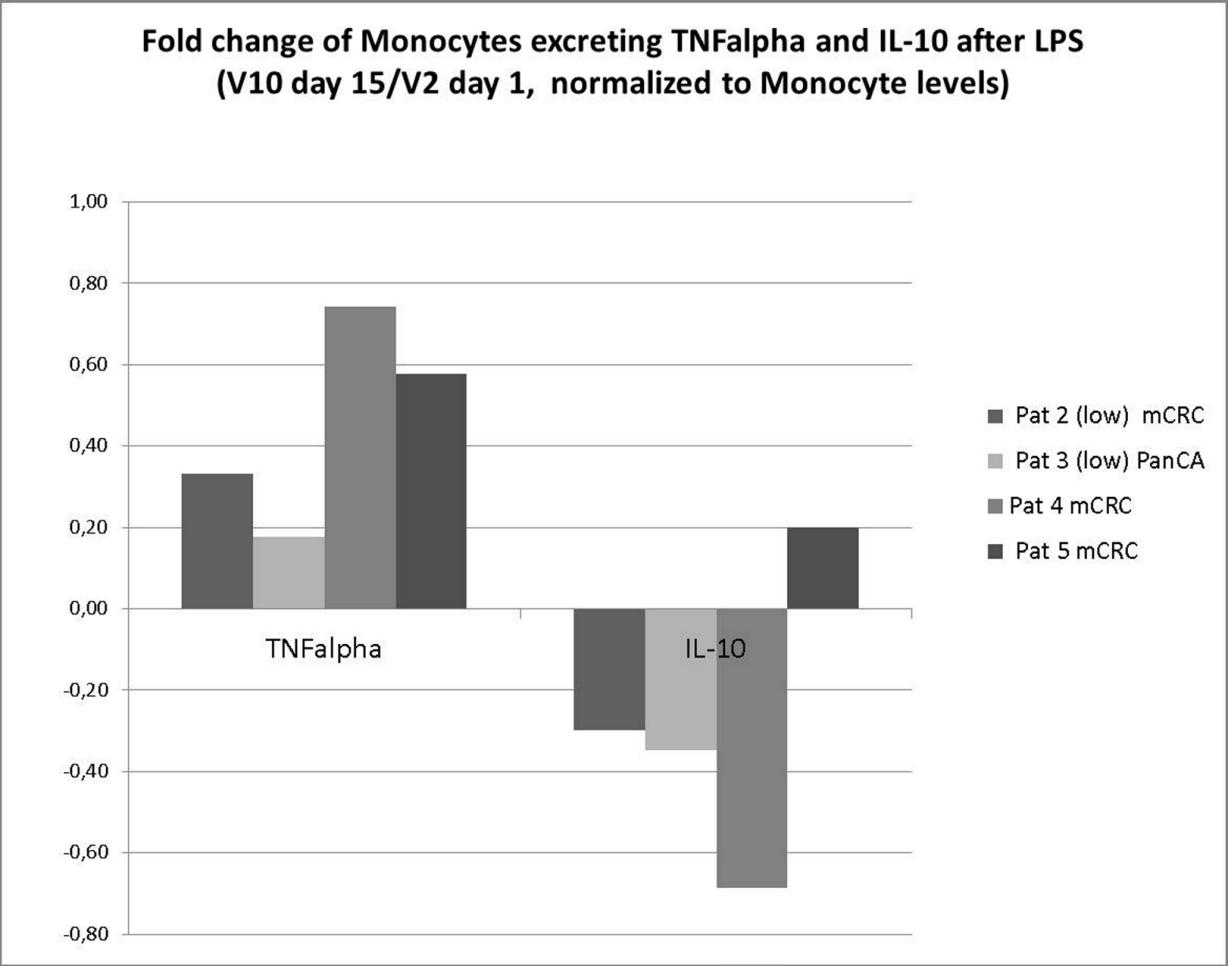
Fold change of Monocytes excreting TNF alpha and IL-10 after Lipopolysaccharide induced cytokine release Enhanced TNF/IL-10 ratio in four patients analyzed showing pro-inflammatory potency (TNF release) of peripheral blood monocytes following TLR-4 stimulation increased over the observation time while the anti-inflammatory potency (IL-10 release) decreased (mean: 3.73 fold, range 1,32 - 6,01 fold).

### Feasibility aspects of the study

Cell-based therapies are not routinely applied in oncology departments and several aspects should be considered as the in-time delivery and application of the cell product with very limited shelf-life < 1h is crucial. The application of genetically modified cells requires adherence to regulatory guidelines.

The donation of autologous bone marrow was performed in the operating room according to tissue donation requirements with a minimum of 12 weeks prior to the intended application. No severe side effects in the pretreated cancer patients were observed. In total, 13 patient-derived bone marrow samples went into the production process and six patients have been treated in the phase I study. In most samples, the amount of starting material was sufficient. A number of products was not released for clinical application (*n* = 5) and 2 pts went into progressive disease and could not be treated.

### Efficacy

An overview of efficacy is given in Table 3. Four of six patients showed stable disease (SD) according to RECIST 1.1 criteria, while 2/6 patients suffered from progressive disease (PD). In patients with SD, the range of change in target lesions was from +5.7% to +18.7%, while it was +21.5% to +31.7% in patients with PD. Evaluation of tumor/serum markers showed heterogeneous values and trends. Median time to progression (TTP) up to 1 year after first MSC_apceth_101 administration was 2.3 months (range, 0.53 to 4.7 months). Median overall survival (OS) up to 1 year after first MSC_apceth_101 administration was 5.61 months (range, 0.97 to 11.47 months). In long-term follow up 2/6 patients lived longer than 12 months. In these patients OS was 16 months and 19 months, respectively.

**Table 3:** Efficacy Parameter Efficacy by RECIST, per cent by change in target lesions baseline to V15, by tumor marker levels, per cent by baseline to V15, TTP and OS in weeks

Patient Number	RECIST	% change target lesion	New non-target lesion	Tumor Marker Change in % Baseline to V15		TTP (months)	OS (months)
1	SD	+ 18.7	None	CEA	+11,7	3.33	4.37
				CA19-9	+88,5		
2	SD	+ 11.3	None	CEA^§^	+33,8	1.57	2.27
				CA19-9^§^	+49,1		
3	SD	+ 8.7	None	CEA	+85,0	2.37	11.47^*^
				CA-19-9	+1401,3		
4	SD	+ 11.1	None	CEA	-11,1	4.7	11.03^*^
				CA-19-9	+0,4		
5	PD	+ 31.7	None	CEA	+52,3	1.27	3.57
				CA-19-9	-23,1		
6	PD	+ 21.5	+1 lung	CEA^§^	+500,7	0.53	0.97
				CA-19-9^§^	+1847,8		

* alive at last visit

^§^ measured at V14

## DISCUSSION

Using genetically modified MSCs in combination with cytotoxic agents is a novel therapeutic approach and may provide a strategy for altering the tumor environment and preventing cancer cell survival.

On the basis of the preclinical efficacy data, the potential clinical application and the medical need we report the results of a first in human, first in class phase I study on the use of genetically modified autologous MSCs in the therapy of heavily pre-treated patients suffering from adenocarcinomas of the GI and HPB tract. To the best of our knowledge, no other clinical trial has been reported on this innovative and newly developed therapeutic approach.

This study explored primarily the safety, tolerability, feasibility and efficacy of MSC_apceth_101 in combination with ganciclovir and met its primary and secondary endpoints.

In general, the safety and tolerability was favorable. Regardless of the dose level, no severe side effects were present during or after IMP infusion. Reported SAEs and SADRs were related to GCV or other underlying diseases. The distinction between complications due to tumor disease *versus* side effects related to study procedures was not possible in all cases, as all patients were heavily pretreated and most of them suffered from extended metastatic disease. If a clear assignment was difficult, the side effects were attributed to the study procedure.

Most importantly and in contrast to effects observed in animal studies, no side effects originating from the use of DMSO, pulmonary trapping and tumor lysis were observed in our patients.

The immunological tests revealed the following two main messages: i) repeated application of gene-modified MSCs, even in combination with GCV, is immunologically safe: no significant signs of systemic inflammatory reaction (except some mild and temporary increase of IL-6), no detectable intratissue (tumor) endothelial activation, no immunogenicity of the cell product, no hints of negative immunomodulation/ -suppression; ii) the therapy induces a switch of the functional properties of monocytes to a more inflammatory phenotype (TNF/IL-10 ratio). These data may suggest a safe and lasting reprogramming of innate immune cells that can enter the target tissue to support tumor destruction by reprogramming stroma cells. However, there are no direct (e.g. *ex vivo* spontaneous IFNg secretion) or indirect (rise of monocytic HLA-DR expression) signs of T-cell activation in response to the therapy, which might be a limitation of the autologous approach. Immune function of patients with cancer following several lines of chemotherapy might be diminished. However, we did not find hints of massive immune deficiency in our tests.

In terms of feasibility, successful development of MSC_apceth_101 for cancer was proven to be particularly challenging. Nevertheless, clinical feasibility was good and the IMP including logistics could be easily and robustly implemented in the clinical routine.

There were no RECIST-type responders observed in this study, 4/6 pts achieved stable disease, while 2/6 suffered from progressive disease. As far as tumor and serum markers were collected, a trend towards stable values during the treatment but rising markers between completion of treatment and post-intervention staging evaluation was notable. The informative value of efficacy data is limited by sample size, heterogeneity and the fact that last line patients partly far beyond standard therapy were recruited for this study.

Furthermore, several aspects limit our results. With six patients suffering from adenocarcinomas of different origins within the GI-tract, our study population is small and heterogeneous. OS and TTP off study-treatment would have been very different. Comparability is therefore limited. In addition, these patients were heavily pretreated with various sequences of therapy. The potential influence of pretreatment on the IMP efficacy cannot be derived from standard preclinical tumor models, as pre-study treatments might have influenced the respective tumor microenvironment in patients. This may interfere with the proposed mode-of-action and may lead to altered MSCs activity.

Pharmacodynamics and pharmacokinetics are difficult to assess in cell-based therapies in contrast to standard therapeutics. Metabolism, distribution and elimination have not been analyzed in this first clinical trial related to the lack of imaging techniques for tracking of the cells and the unclear metabolic pathway of the infused cells.

Time intervals for the scheduling of MSCs and GCV administration was based on the investigators preclinical studies, where this application routine has proven optimal and feasible for ensuring that MSCs have enough time to enter the tumor stroma from the peripheral circulation and activate the therapeutic transgene [12, 15–17]. However, further investigations need to prove the optimal sequence of this procedure. Additional fields of research may include strategies to enhance the number of therapeutic cells homing to tumors and improvement of the tissue specific GDEPT construct.

From our present experience with 6 patients and with no dose limiting toxicity present, no conclusive linear dose-response relationship can be concluded. As the IMP was dosed according to body weight, overlapping absolute doses have been applied. Higher doses in three patients did not lead to worse tolerability or better tumor response. However, comparison of both treatment groups does not allow direct comparison of clinical efficacy between cohorts since recruitment was not randomized nor was the trial prospectively powered to make such a comparison. Dose-escalation was performed but no dose-limiting toxicity was documented. It needs to be pointed out that the low and high dose differed only by 2-fold. Therefore, it is conceivable that the chance of detecting consistent differences with regard to toxicity or efficacy in the groups was low. Using larger dose escalation steps was hampered by the autologous nature of the applied MSCs product. The starting material for the generation of the MSCs product was autologous bone morrow collected from heavily pretreated cancer patients. During the implementation of the manufacturing process it was found that this type of starting material is limited with regards to the number of MSCs which can be produced from it and is associated with increased out-of-specification results in quality control. For this technical reason, larger dose escalation steps could not be included into the trial protocol.

In conclusion, this innovative and newly developed stem-cell based therapeutic approach is safe, tolerable, and feasible. To the best of our knowledge, this study shows the first successful and safe transfer of the MSC-based cancer therapy in the clinical setting. All relevant regulatory requirements for cell- and gene therapy products (ATMPs) were fulfilled. Tumor response has to be evaluated critically and put into perspective with pre-treatment, patient- and tumor- status and the, yet, unknown optimal dose. As preclinical data suggested better response at high doses, the recommended phase II dose derived from this study is the higher total dose of 3 × 10^6^ cells/kg. Further optimization of the clinical study protocol by definition of patient's condition and the application regimen should precede the transition of MSC_apceth_101 into the next phase of clinical development. This should include the selection of the most appropriate transgenes and vectors, a deeper understanding of how to best combine MSC_apceth_101 with other (immuno-) therapies, as well as a better understanding regarding the optimal sequence and schedule of this novel therapeutic approach.

## MATERIALS AND METHODS

### Study design

The safety and tolerability of the IMP was investigated in six patients in a standard 3 + 3 dose escalation design [25]. Three of the six patients received the cells at a total dose of 1.5 × 10^6^ cells/kg, applied intravenously in three equal doses of 0.5 × 10^6^ cells/kg each one week apart. The other three patients received up to three doses of 1 × 10^6^ cells/kg adding up to a total dose of 3 × 10^6^ cells/kg after obtaining approval for dose escalation by the independent DSMB (Data and Safety Monitoring Board). Injection of the IMP was followed by intravenous GCV injection dosed according to the manufacturer's recommendation, which was at 5 mg/kg b.i.d. when a creatinine clearance of greater than 69 ml/min was present and 2.5 mg/kg b.i.d. if the creatinine clearance was 50-69 ml/min. Time interval between GCV administrations was 12 ± 3 hours. GCV was given on three consecutive days starting between 48-72 hours after injection of MSC_apceth_101. The rationale for the scheduling of MSC and GCV administration was based on the investigators preclinical studies, where these time intervals have proven optimal for ensuring that MSCs have enough time to enter the tumor stroma from the peripheral circulation and to activate the therapeutic transgene [15–17]. The study design was published previously and is displayed in Figure 1 [12].

### IMP manufacturing

Patients were screened for bone marrow donation according to the German hemotherapy guidelines and the German transplantation law. MSCs were obtained for each individual patient by bone marrow aspiration performed according to a protocol approved by the responsible ethics committee in addition to the clinical trial protocol. The IMP was produced under xeno-free conditions according to Good Manufacturing Practice (GMP) guidelines. Expanded MSCs were transduced with a gamma-retroviral vector (“GMP grade”, provided by EUFETS, Idar-Oberstein, Germany) containing the HSV-TK transgene and a puromycin resistance gene. Transduced cells were selected with puromycin and further expanded to reach the clinical dose. The final formulation consisted of genetically modified MSCs in Dimethyl sulfoxide (DMSO) and Hydroxyethyl starch (HAES) at concentrations of 2×10^6^ /ml or 5×10^6^ / ml. The cells were cryopreserved under controlled conditions. Final lots were tested for viability, cell identity, purity, vector copy number, transgene expression, GCV sensitivity, viral pathogens, microbiologic contamination, mycoplasma and endotoxin for release according to specifications. The manufactured MSCs confirm to the specifications laid out in the position paper by Dominici et al. [9]. MSC_apceth_101 was manufactured and provided by apceth GmbH & Co.KG (Munich, Germany). The manufacturing process of the autologous product MSC_apceth_101 has also been described elsewhere [12].

### IMP administration

The handling of the product was performed according to the processing of hematopoietic stem cell transplants such as cord blood units. MSC_apceth_101 was thawed at a temperature of 37°C ± 1°C immediately before infusion. The time between thawing of MSC_apceth_101 and administration to the patient was not to exceed 45 minutes. No co-infusion was permitted.

After premedication (antihistamines H1- and/or H2-blocker i.v.), MSC_apceth_101 was administered by intravenous infusion over a period of up to 15 minutes (1-2 bags).

### Drug supply

MSC_apceth_101 was produced and provided by apceth GmbH and Co KG (Munich, Germany). Ganciclovir was provided as an approved drug product by the local pharmacy and dosed as described in the Summary of Product Characteristics of Cymeven^®^ i.v..

### Study eligibility criteria

We enrolled adult patients (age ≥ 18 years) with histologically confirmed adenocarcinoma of the gastrointestinal tract refractory to standard therapy. In addition premature or scheduled termination of standard therapy due to intolerability/progress/inefficacy or no acceptance by the patient was an inclusion criterion. Disease status defined by RECIST, version 1.1, an Eastern Cooperative Oncology Group (ECOG) performance status score of ≤ 2 and adequate organ function, including liver, kidney and bone marrow function were further inclusion criteria. Exclusion criteria included severe heart and lung diseases, symptomatic peritoneal carcinomatosis (e.g. by the presence of ascites), symptomatic pleural or pericardial effusion, serious uncontrolled acute infections less than three weeks before visit 1, Patients with HSV-, HCV- and/or HIV infections, Immunodeficiency or systemic autoimmune diseases or known malformations of the GI-tract, use of any immunomodulators, recent need for chemotherapy or radiotherapy or cytokine treatment (e.g. interferons, G-CSF or GM-CSF) within 2 weeks before IMP infusion or anticipation of chemo- or radiotherapy during treatment with MSC_apceth_101 and GCV until 2 days after the last administration of GCV, patients requiring corticoids in doses above the Cushing threshold and any surgery in the last four weeks before the administration of MSC_apceth_101.

### Safety relevant data

Safety relevant data were serious and non-serious adverse events, including any clinically relevant changes of laboratory parameters or vital signs of patients. The safety data was prepared for the Data and Safety Monitoring Board (DSMB) for review. The safety data along with the recommendation of the DSMB was forwarded to the Paul-Ehrlich-Institute, the German national regulation authority for IMP trials, which is responsible for the evaluation of somatic and cell therapy products, and the leading ethics committee as a substantial amendment.

Special attention was paid to product specific side effects and safety signals, respectively. With regard to the use of DMSO and GCV as well as the possible tumor lysis syndrome a wide range of laboratory blood work, such as LDH, kidney function tests and changes in complete blood count were monitored. Potential pulmonary trapping was followed by measuring the peripheral oxygen saturation before, 15, 30 and 60 min and 6 (+/-1 h) hours and 24 (+/-2 h) hours after the IMP infusion to ensure an unchanged lung function by means of a standard pulse oximetry. Quality of life (QoL) was evaluated by QLQ-C30 questionnaire.

### Immunological biomarkers

We selected well established and validated tests which are able to answer the questions we addressed as seen in previous studies in variety of patient studies. To evaluate the systemic inflammatory response, we analyzed the plasma levels of TNF, IL-6, IL-10, and total IL-8 blood levels (after erythrocyte lysis) by standard ELISA techniques (Immulite, Siemens) [26–28]. For checking putative intratissue (tumor) endothelial activation, sE-Selectin total (whole blood) IL-8 was measured. The *ex vivo* Lipopolysaccharide (LPS)-induced TNF/IL-10 cytokine release was used to analyze the inflammatory/ anti-inflammatory potency (M1/M2)of circulating monocytes (Immulite). Monocytic HLA-DR expression as sensitive marker for monitoring global Th1-cell activation, was quantified by flowcytometry using HLA-DR Quantribrite (Becton-Dickinson). To test the immunogenicity of gene modified autologous MSCs, T-cell reactivity was monitored by 24h *ex vivo* stimulation of patients T-cells with the MSCs and IFNg-Elispot (AID) as read-out.

### Efficacy

Efficacy was evaluated using tumor response data, measured by (a) total and individual size of local relapse and metastases by CT or MRT according to RECIST 1.1 criteria and tumor and serum markers, (b) time to progression (TTP) up to 1 year after first MSC_apceth_101 administration and (c) overall survival (OS) up to 1 year after first MSC_apceth_101 administration.

OS and TTP were calculated starting from the first day of MSCs administration (visit 2). Censoring was performed 12 months after first MSCs administration (visit 18), or at the date of death when applicable.

## Abbreviations

AE: Adverse Event
ATMP: Advanced Therapy Medicinal Products
b.i.d: bis in die - twice a day
CCC: cholangiocarcinoma
CT: Computed Tomography
dl: deciliter
DMSO: Dimethyl sulfoxide
DNA: deoxyribonucleic acid
DSMB: Data and Safety Monitoring Board
EC: endothelial cell
ECOG: Eastern Cooperative Oncology Group
G-CSF: granulocyte-colony stimulating factor
GCV: Ganciclovir
GDEPT: Gene-directed enzyme-producing therapy
GI: gastrointestinal
GM-CSF: granulocyte macrophage colony-stimulating factor
GMP: good manufacturing practice
HAES: hydroxyethyl starch
HCV: hepatitis C
HIV: human immunodeficiency virus
HLA-DR: Human Leukocyte Antigen - antigen D Related
HSV: herpes simplex virus
HSV-TK: herpes-simplex-virus thymidine kinase
HPB: Hepato-Pancreato-Biliary
IFN: Interferone
IL: interleukin
IMP: Investigational Medicinal Product
i.v.: intravenous
kg: kilogram
mCRC: metastatic colorectal cancer
mg: milligram
MSC: Mesenchymal Stromal Cells
MRI: Magnetic Resonance Imaging
OS: overall survival
PanCa: Pancreatic cancer
PD: progressive disease
QoL: Quality of life
RECIST: Response Evaluation Criteria In Solid Tumors
SADR: severe adverse drug reaction
SAE: Serious Adverse Event
SD: stable disease
SmPC: summary of product Characteristics
TAF: tumor-associated fibroblasts
TNF: tumor necrosis factor
TLR: Toll-like Receptor
TTP: time to progression
